# Predicting Care Needs in Community-Dwelling Older Adults Using Explainable Machine Learning and a Multidimensional Approach Integrating Health, Social, and Environmental Factors: Cross-Sectional Study

**DOI:** 10.2196/93371

**Published:** 2026-07-31

**Authors:** Hyeri ‍Shin

**Affiliations:** 1 AgeTech-Service Convergence Major, Department of Gerontology, Kyung Hee University, 1732, Deogyeong-daero, Giheung-gu, Yongin-si, Gyeonggi-do, 17104, Republic of Korea, 82 312012940

**Keywords:** older adults, care needs, long-term care, machine learning, explainable AI

## Abstract

**Background:**

Rapid population aging and a worsening shortage of care workers necessitate the identification of older adults who require proactive interventions. Although machine learning (ML) has been increasingly applied in gerontology, existing studies have predominantly focused on social isolation, loneliness, depression, falls, and frailty in isolation rather than on the integrated construct of care needs.

**Objective:**

This study aimed to develop and interpret an explainable ML model that identifies care needs in community-dwelling Korean older adults. Beyond physical health indicators such as disease and functional status, this study adopted a comprehensive approach that included mental health, cognitive function, health behaviors, and socioenvironmental determinants, such as social participation, social support, and the housing environment, to present an integrated model encompassing both health and social care needs.

**Methods:**

Data were obtained from the 2023 Korea Senior Survey, a nationally representative sample of 10,078 community-dwelling adults aged 60 years and older. The data were split 70:30 into training (n=7054) and held-out test (n=3024) sets. Seven algorithms were compared (logistic regression, decision tree, support vector machine, random forest, gradient-boosted decision trees, extreme gradient boosting, and light gradient boosting machine) using stratified 5-fold cross-validation on the training set. Discrimination was assessed by the area under the receiver operating characteristic curve (AUC). Model interpretability used Shapley additive explanations with bootstrap stability assessment across folds.

**Results:**

Model A (excluding activities of daily living or instrumental activities of daily living [IADL]) achieved good discrimination (AUC 0.892, 95% CI 0.866‐0.918), adequate calibration (calibration slope=0.826), and positive clinical net benefit, demonstrating that upstream factors alone can identify older adults with care needs without relying on functional status. Shapley additive explanations analysis identified age, nutritional risk, employment status, depressive symptoms, self-rated health, cognitive function, household income, and home modification as the leading predictors, with high rank stability across cross-validation folds. Model B (including activities of daily living or IADL) yielded a higher AUC (0.976, 95% CI 0.962‐0.989), but this reflected the near-tautological relationship between IADL and self-reported care needs rather than genuine upstream predictive value. Using an objective composite outcome yielded equivalent discrimination (AUC 0.892), supporting robustness to the outcome definition.

**Conclusions:**

Explainable ML models offer high predictive accuracy and strong interpretability for identifying care needs among older adults. Care needs in community-dwelling Korean older adults can be identified with good discrimination, calibration, and clinical net benefit using multidimensional nonfunctional factors alone. By highlighting the significant roles of health, social, and environmental factors, this study provides empirical evidence to support evidence-based decision-making for the Long-Term Care Insurance system and integrated community care policies.

## Introduction

The intersection of rapid demographic aging and a shrinking care workforce in South Korea creates an urgent need to identify older adults who currently require care or are at elevated risk. In response to these demographic changes, South Korea introduced the Long-Term Care Insurance (LTCI) system in 2008. As shortages in the care workforce intensify, the Korean government is preparing to implement the Act on Integrated Support for Community Care (Integrated Community Care Act) in 2026. These policy initiatives aim to provide a comprehensive range of services, from mild support to intensive care, enabling older adults to maintain their lives within their communities. With the enactment of this legislation, it is essential to predict care needs across multiple dimensions; however, current eligibility assessments predominantly rely on functional status, such as activities of daily living (ADL). To effectively implement integrated care across the continuum from mild to severe needs, it is necessary to move beyond simple functional assessments and analyze predictive factors from a multidimensional perspective using big data.

Although machine learning (ML) has been increasingly applied in gerontology to address these complexities, existing studies have tended to be thematic rather than holistic. Research in this field has predominantly focused on social isolation and loneliness. For instance, ML has been used to predict social isolation among older adults in China [1] and sleep disorders among older adults with multimorbidity [2]. Similarly, domestic research in Korea has largely focused on specific geriatric syndromes, such as depression [3,4] and fall risk [5]. While valuable, these studies address isolated aspects of aging rather than the comprehensive demand for care. Recent applications of explainable ML to other geriatric outcomes have included cognitive decline [6] and frailty classification [7] in community-dwelling older adults, but none have focused on the integrated construct of care needs.

Research specifically predicting the care needs of older adults remains limited. Few studies have been conducted to predict care needs in Japan [8] and Korea [9] using public insurance claims data. However, these studies are limited by their reliance on administrative data, including medical history, health care, and long-term care service usage records, and do not consider sociodemographic and environmental factors that significantly influence individual care needs. Moreover, some studies have predicted physical disability based on ADL and instrumental activities of daily living (IADL) scores [10], as well as frailty [11]. A conceptual gap exists between functional disability, which is generally defined as a clinical measure, and perceived care needs, which are defined as a subjective demand for assistance [12].

Although the volume of multidimensional ML research in gerontology is expanding, there is a notable absence of studies applying these techniques to predict comprehensive care needs among older Korean adults using survey data. To address this gap, this study has 2 objectives. The first is to develop and interpret an explainable ML model that identifies community-dwelling older adults with care needs using multidimensional upstream factors alone, excluding functional status (ADL and IADL). The second is to characterize the relative contribution of predictors across demographic, health, behavioral, psychological, cognitive, socioeconomic, social support, and environmental domains, thereby examining whether care needs behave as a multidimensional construct. This study aims to provide a robust evidence base for the successful implementation of upcoming integrated care policies.

## Methods

### Data Source and Participants

This study used data from the 2023 Korea Senior Survey, a nationally representative survey initiated in 2007, to examine the characteristics of older adults in Korea. This survey collects comprehensive information across multiple domains, including health status, economic conditions, and social participation, and enables monitoring of changes over time.

A total of 10,078 community-dwelling adults aged 60 years and older were included in this analysis. Data on demographic, health-related, functional, and psychosocial characteristics were extracted for this study. Outcome prevalence was 8.7% (875/10,078, 95% CI 8.1%‐9.3%), giving an imbalance ratio of approximately 1:10.5.

Missingness was very low across the candidate predictor set (median 0.0%, IQR 0.0%‐0.0% per variable; range 0.0%‐10.6%). Two variables exceeding the prespecified 10% threshold, frequency of contact with children and frequency of contact with friends, were excluded a priori. Continuous variables were imputed using the median and categorical variables using the mode, with imputation parameters fitted only on the training fold and applied to held-out folds to prevent leakage. A complete-case sensitivity analysis yielded substantively identical results.

This study was reported in accordance with the TRIPOD+AI (Transparent Reporting of a Multivariable Prediction Model for Individual Prognosis or Diagnosis+AI) statement [13]. The completed checklist is provided in Checklist 1.

### Ethical Considerations

The sampling design and survey content of the 2023 National Survey of Older Koreans were officially approved by Statistics Korea (National Statistics Approval Number 117071). The study protocol was reviewed and approved by the Institutional Review Board of the Korea Institute for Health and Social Affairs (2023‐078).

### Measures

In this study, care needs were assessed using a self-reported questionnaire. Participants were asked whether they required assistance or care in their daily lives. Responses were dichotomized to define the outcome variable. Participants who reported requiring care were classified as the care-needs group and assigned a value of 1, whereas those who reported not requiring care were classified as the no–care-needs group and assigned a value of 0.

Based on previous studies and established theoretical frameworks on care needs among older adults, a comprehensive set of multidimensional predictive variables was selected. Demographic characteristics included gender, age, marital status (spouse), educational attainment, current employment status, number of household members, and household income. Subjective health variables comprised self-rated health and spouse’s self-rated health. Physical health-related variables included the number of chronic conditions, BMI, sleep quality, and history of falls. Health behavior variables included current smoking status, current drinking status, and regular exercise. Nutritional health was assessed using nutritional risk indicators, including nutritional risk status and the total Nutritional Screening Initiative (NSI) score.

Health care utilization variables included outpatient or hospital visits and health-screening experience within the past 2 years. Mental and cognitive status was measured using depressive symptoms and cognitive function. Functional status was evaluated using ADL and IADL total scores. Binary limitation indicators were excluded from the candidate predictor set to avoid redundancy with the continuous totals. Social participation variables encompassed participation in leisure activities, educational activities, hobby clubs, social groups, political groups, volunteer activities, religious affiliations, and the use of senior welfare centers. Social support was assessed based on the number of available supporters when feeling depressed, when ill, or when needing to borrow money. Finally, living environment factors included housing satisfaction, home modification level, and area of residence.

Composite social-contact indices that overlapped with the outcome construct were excluded a priori as leakage candidates. From the 87 original variables, 6 were excluded a priori: 2 due to high missingness (more than 10%), 2 as redundant binary indicators of functional limitations, and 2 as leakage candidates. This yielded 81 final candidate predictors—79 were used in model A (excluding ADL and IADL totals) and 81 in model B.

### Analytical Strategy

Two prespecified models were compared. Model A (primary, upstream) included all 79 candidate predictors except the ADL and IADL totals. This model targets the practical task of identifying older adults at risk before functional dependence is established, the relevant operating point for preventive screening under the Integrated Community Care Act. Model B (benchmark, full) included all 81 candidate predictors and is reported only as a benchmark to characterize the upper bound of predictive performance when functional status is observable.

The analytical process for both models proceeded through descriptive analysis, predictive modeling, feature selection, class-imbalance handling, hyperparameter specification, validation, calibration and decision-curve analysis, model interpretation, and sensitivity analyses.

First, descriptive analyses were conducted to compare the baseline characteristics of older adults with and without care needs. Independent-sample *2-tailed t* tests were used for continuous variables, and chi-square tests were applied for categorical variables.

Second, for predictive modeling, the dataset containing 10,078 samples was split 70:30 (stratified by outcome) into a training set (prevalence, 612/7054, 8.7%) and a held-out test set (prevalence, 263/3024, 8.7%). Seven ML algorithms were compared: logistic regression (LR), decision tree (DT), support vector machine (SVM), random forest (RF), gradient-boosted decision trees (GBDT), extreme gradient boosting (XGBoost), and light gradient boosting machine (LightGBM).

Third, all candidate predictors were retained for model fitting; built-in regularization within each algorithm provided implicit feature weighting (L2 penalty for LR, tree-based split importance for RF and gradient-boosted models, a radial basis function kernel with C=1.0 for SVM, and depth-limited splits for the DT). To address potential redundancy across the candidate set, post hoc Shapley additive explanation (SHAP)-based feature ranking with bootstrap-based stability assessment across cross-validation folds (Jaccard similarity of the top-10 set) was used to characterize the relative contribution of individual predictors (Multimedia Appendix 1).

Fourth, the class imbalance (1:10.5) was handled using 2 complementary approaches. For boosted models (XGBoost, LightGBM, and GBDT), a class-weighted loss function was used, with the positive class weighted by the inverse of its frequency. For nonboosted models, the synthetic minority oversampling technique [14] was applied only within each cross-validation training fold, never on validation or test partitions, to prevent information leakage. LR and SVM models were additionally fitted with class-balanced weights as a robustness check.

Fifth, hyperparameters were prespecified for each algorithm based on values commonly used in the ML literature for moderately sized clinical datasets; no algorithm-specific hyperparameter optimization was performed (XGBoost: 400 estimators, learning rate 0.05, max depth 4, subsample 0.8, colsample_bytree 0.8; LightGBM: 400 estimators, learning rate 0.05, max depth 6, num_leaves 31; GBDT: 300 estimators, learning rate 0.05, max depth 3; RF: 500 trees, min_samples_leaf 2; LR: L2 penalty with C=1.0; decision tree: max depth 6, min_samples_leaf 20; SVM: RBF kernel with C=1.0). Boosted models used positive-class scale weights computed from each training fold; nonboosted models used class-balanced weights (with synthetic minority oversampling technique applied within the cross-validation training fold for nonboosted models). The random seed was set to 42 for reproducibility. All analyses were conducted in Python 3 on Google Colab using *scikit-learn*, *statsmodels*, *SciPy*, *LightGBM*, *XGBoost*, *imbalanced-learn*, and *SHAP*.

Sixth, the validation strategy combined stratified 5-fold cross-validation on the training portion (with the mean, SD across folds reported as the primary performance estimate) with a final evaluation on the held-out test set. Discrimination was assessed using the area under the receiver operating characteristic curve (AUC), with 95% CIs from the DeLong method on the test fold and from 1000 bootstrap resamples. Additional metrics included the precision-recall AUC (PR-AUC), the prevalence-adjusted relative PR-AUC (PR-AUC ÷ outcome prevalence), sensitivity, specificity, the *F*_1_-score, and the Matthews correlation coefficient (MCC). Performance was reported at 3 operating thresholds, the default of 0.5, prevalence-matched (0.087), and Youden-optimal, to demonstrate the sensitivity-specificity trade-off across the relevant decision range.

Seventh, calibration was assessed on the held-out test set using the Brier score, calibration slope and intercept, and reliability diagrams in which predicted probabilities were grouped into 10 quantile-based bins. Predicted probabilities were post hoc recalibrated using isotonic regression fitted on the training portion via 5-fold cross-validated stacking. Decision-curve analysis was performed across threshold probabilities ranging from 1% to 50% [15] to assess clinical net benefit relative to the “treat all” and “treat none” reference strategies.

Eighth, model interpretability was assessed using SHAP [16]. SHAP values were computed within each of the 5 cross-validation folds, and the mean |SHAP| values were computed with bootstrap-derived 95% CIs, together with the rank stability of the top-10 features quantified by the Jaccard similarity index across folds (Multimedia Appendix 1).

Finally, prespecified sensitivity analyses included leave-one-feature-out for the top-10 predictors of model A; an alternative outcome operationalization using an objective composite (2 or more IADL limitations and/or 1 or more ADL limitations; prevalence, 1411/10,078, 14.0%); stratification of performance by depressive-symptom quartile; and complete-case analysis. Discrimination was preserved across Center for Epidemiologic Studies Depression Scale–Short Form (CES-D-SF) score quartiles (AUC range 0.86‐0.91), arguing against differential model performance driven by depression-related self-report bias. Complete-case analysis yielded substantively identical results.

## Results

### Sample Characteristics

Table 1 presents the baseline characteristics of older adults according to the care-needs status. Compared with older adults without care needs, those with care needs were significantly older and more likely to be female older adults. They were also less likely to have a spouse and had substantially lower levels of educational attainment and employment participation. Household income was significantly lower in the care-needs group, whereas the number of household members did not differ significantly between the 2 groups. Regarding health status, older adults with care needs reported poorer self-rated health and poorer spouse’s self-rated health, and they had a significantly higher number of chronic conditions. BMI was slightly lower in the care-needs group. Regarding social resources, the care-needs group had fewer available supporters when feeling depressed, when ill, or when needing to borrow money, indicating lower levels of social support.

**Table 1.** Respondents’ demographic profile (N=10,078).

Variable	Older adults without care needs (n=9203)	Older adults with care needs (n=875)	*P* value
Demographics			
Sex, n (%)			
Female	5620 (61.1)	586 (67.0)	<.001
Male	3583 (38.9)	289 (33.0)	<.001
Age (y), mean (SD)	73.48 (6.43)	80.97 (6.90)	<.001
Spouse, n (%)			
No	3617 (39.3)	510 (58.3)	<.001
Yes	5586 (60.7)	365 (41.7)	<.001
Educational attainment, n (%)			
Elementary school or less	3820 (41.5)	629 (71.9)	<.001
Middle school	2003 (21.8)	124 (14.2)	<.001
High school	2777 (30.2)	99 (11.3)	<.001
College or higher	603 (6.6)	23 (2.6)	<.001
Currently employed, n (%)			
No	5339 (58.0)	791 (90.4)	<.001
Yes	3864 (41.9)	84 (9.6)	<.001
Number of household members, mean (SD)	1.76 (0.66)	1.72 (0.77)	.19
Household income (KR ₩, thousand)^a^, mean (SD)	3341.14 (3489.78)	2311.49 (2260.18)	<.001
Subjective health, mean (SD)			
Self-rated health	3.27 (0.92)	2.97 (2.21)	<.001
Spouse self-rated health	5.54 (2.86)	6.31 (3.24)	<.001
Physical health, mean (SD)			
Number of chronic conditions	2.08 (1.51)	3.57 (2.04)	<.001
BMI (kg/m²)	23.68 (2.58)	23.06 (3.09)	<.001
Sleep quality	3.41 (0.88)	3.50 (2.06)	.19
Falls			
History of falls (past year), n (%)			
No	8730 (94.9)	709 (81.0)	<.001
Yes	473 (5.1)	166 (18.9)	<.001
Health behavior			
Current smoking, n (%)			
No	8436 (91.7)	831 (94.9)	<.001
Yes	767 (8.3)	44 (5.0)	<.001
Current drinking, n (%)			
No	5970 (64.9)	748 (85.5)	<.001
Yes	3233 (35.1)	127 (14.5)	<.001
Regular exercise, n (%)			
No	4141 (44.9)	561 (64.1)	<.001
Yes	5062 (55.0)	314 (35.9)	<.001
Nutritional health			
Nutritional risk (NSI)^b^, n (%)			
Normal	6356 (69.1)	246 (28.1)	<.001
At risk	2847 (30.9)	629 (71.9)	<.001
Nutritional risk total score, mean (SD)	2.02 (2.32)	4.57 (3.27)	<.001
Health care utilization			
Outpatient or hospital visit, n (%)			
No	2952 (32.1)	144 (16.5)	<.001
Yes	6251 (67.9)	731 (83.5)	<.001
Health screening (past 2 years), n (%)			
No	1861 (20.2)	297 (33.9)	<.001
Yes	7342 (79.8)	578 (66.1)	<.001
Mental or cognitive health, mean (SD)			
Depressive symptoms	3.34 (8.78)	19.69 (3.49)	<.001
Cognitive function	24.87 (4.50)	20.28 (5.70)	<.001
Functional status			
ADL^c^ limitation, n (%)			
No	8902 (96.7)	356 (40.7)	<.001
Yes	301 (3.3)	519 (59.3)	<.001
ADL (total score), mean (SD)	7.07 (0.54)	8.94 (2.89)	<.001
IADL^d^ limitation, n (%)			
No	8317 (90.4)	2 (0.2)	<.001
Yes	886 (9.6)	873 (99.8)	<.001
IADL (total score)	10.32 (1.46)	16.51 (5.65)	<.001
Social participation			
Participation in leisure activities, n (%)			
No	1499 (16.3)	284 (32.5)	<.001
Yes	7704 (83.7)	591 (67.5)	<.001
Participation in educational activities, n (%)			
No	7886 (85.7)	795 (90.9)	<.001
Yes	1317 (14.3)	80 (9.1)	<.001
Participation in hobby clubs, n (%)			
No	8587 (93.3)	857 (97.9)	<.001
Yes	616 (6.7)	18 (2.1)	<.001
Participation in social groups, n (%)			
No	4050 (44.0)	727 (83.1)	<.001
Yes	5153 (55.9)	148 (16.9)	<.001
Participation in political groups, n (%)			
No	9065 (98.5)	869 (99.3)	.07
Yes	138 (1.5)	6 (0.7)	.07
Volunteer experience, n (%)			
No	8937 (97.1)	872 (99.7)	<.001
Yes	266 (2.9)	3 (0.3)	<.001
Religious affiliation, n (%)			
No	5438 (59.1)	540 (61.7)	.14
Yes	3765 (40.9)	335 (38.3)	.14
Use of senior center, n (%)			
No	6679 (72.6)	551 (62.9)	<.001
Yes	2524 (27.4)	324 (37.0)	<.001
Use of senior welfare center, n (%)			
No	8328 (90.5)	793 (90.6)	.94
Yes	875 (9.5)	82 (9.4)	.94
Social support, mean (SD)			
Number of available supporters when feeling depressed	2.41 (1.68)	1.77 (1.65)	<.001
Number of available supporters when ill	1.78 (1.32)	1.43 (1.27)	<.001
Number of available supporters to borrow money from	1.15 (1.21)	0.74 (1.15)	<.001
Living environment, mean (SD)			
Housing satisfaction	3.78 (0.71)	4.20 (1.78)	<.001
Home modification level, n (%)			
None	2860 (31.1)	167 (19.1)	<.001
Low	3286 (35.7)	263 (30.1)	<.001
Mid	2447 (26.6)	337 (38.5)	<.001
High	610 (6.6)	108 (12.3)	<.001
Area of residence, n (%)			
Rural	2696 (29.3)	321 (36.7)	<.001
Urban	6507 (70.7)	554 (63.3)	<.001

a Monthly household income was measured at KR ₩10,000=US $7.67 as of November 10, 2023.

b NSI: Nutritional Screening Initiative.

c ADL: activities of daily living.

d IADL: instrumental activities of daily living.

### Variable Diagnostics

Variance inflation factors (VIFs) were computed for all numeric predictors in model A. The highest VIF was 2.31 (number of available supporters when ill), well below the conventional concerning threshold of 5 and even below the conservative threshold of 2.5. The 7 highest-VIF variables (all VIFs<2.5) reflect expected modest correlations among related health and social-support constructs, but each carries sufficient independent information to be retained. No predictor required removal on multicollinearity grounds.

### Multivariable LR

As a confirmatory descriptive complement to the ML analyses, Table 2 presents the results of the multivariable LR analysis identifying independent factors associated with care needs among older adults.

**Table 2.** Multivariate stepwise logistic regression analysis of care needs^a^.

Variable	β^b^ (SE)	OR^c^ (95% CI)	*P* value
Age	0.068 (0.008)	1.07 (1.05‐1.09)	<.001
Number of household members	0.076 (0.092)	1.08 (0.90‐1.29)	.41
Household income	0.000 (0.000)	1.00 (1.00‐1.00)	.72
Self-rated health	−0.455 (0.071)	0.63 (0.55‐0.73)	<.001
Spouse self-rated health	0.046 (0.020)	1.05 (1.01‐1.09)	.02
Sleep quality	−0.041 (0.066)	0.96 (0.84‐1.09)	.54
Hypertension	0.050 (0.116)	1.05 (0.84‐1.32)	.67
Hyperlipidemia	−0.148 (0.111)	0.86 (0.69‐1.07)	.19
Diabetes mellitus	0.158 (0.108)	1.17 (0.95‐1.45)	.14
Osteoporosis	0.395 (0.127)	1.48 (1.16‐1.91)	.002
Dementia	0.571 (0.260)	1.77 (1.06‐2.94)	.03
BMI	0.019 (0.017)	1.02 (0.99‐1.05)	.26
Nutritional risk (total score)	0.096 (0.018)	1.10 (1.06‐1.14)	<.001
Depressive symptoms	0.037 (0.017)	1.04 (1.00‐1.07)	.03
Cognitive function	−0.027 (0.011)	0.97 (0.95‐0.99)	.02
Activities of daily living	−0.218 (0.059)	0.80 (0.72‐0.90)	<.001
Instrumental activities of daily living	0.496 (0.026)	1.64 (1.56‐1.73)	<.001
Number of available supporters when feeling depressed	0.006 (0.043)	1.01 (0.93‐1.09)	.89
Number of available supporters when ill	0.056 (0.058)	1.06 (0.94‐1.18)	.33
Number of available supporters to borrow money from	−0.102 (0.055)	0.90 (0.81‐1.00)	.06
Housing satisfaction	0.254 (0.079)	1.29 (1.11‐1.50)	.001
Home modification level	0.056 (0.024)	1.06 (1.01‐1.11)	.02

a Observations=9951; Pseudo-R2=0.4432; log-likelihood=−1528.1, LL-null=–2744.4; and LLR *P*<.001.

b β: regression coefficient.

c OR: odds ratio.

Older age was significantly associated with higher odds of care needs, indicating an increased likelihood of requiring care with advancing age. In contrast, better self-rated health was associated with lower odds of care needs, suggesting a protective effect. Regarding health-related factors, spouse’s poor self-rated health, osteoporosis, and dementia were significantly associated with increased odds of care needs. Additionally, higher nutritional risk scores and greater depressive symptoms were positively associated with care needs, indicating that nutritional vulnerability and mental health status play important roles.

Functional status showed strong and distinct associations. Greater limitations in IADL were associated with higher odds of care needs, whereas better performance in basic ADL was associated with lower odds. Higher cognitive function scores were also inversely associated with care needs. Regarding the residential environment, higher housing satisfaction and home modifications were significantly associated with increased odds of care needs, suggesting that housing conditions and environmental adaptations reflect underlying care demands.

Overall, care needs among older adults were independently associated with advanced age, poor health and nutritional status, mental and cognitive vulnerability, functional impairment, and housing-related factors.

**Figure 1.**
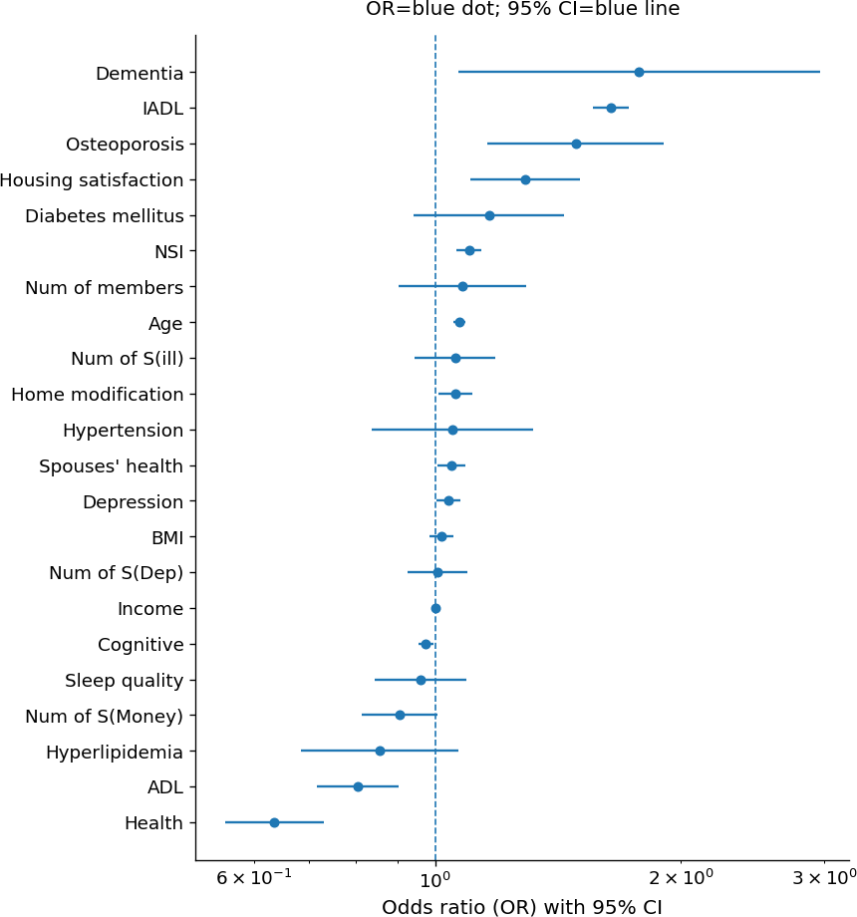
Various factors are associated with the specific care needs of the population. ADL: activities of daily living; IADL: instrumental activities of daily living; NSI: Nutritional Screening Initiative total score; Num of members: number of household members; Num of S(Dep): number of supporters when feeling depressed; Num of S(ill): number of supporters when ill; Num of S(Money): number of supporters to borrow money from; Income: household income; Health: self-rated health; Spouses’ health: spouses’ self-rated health.

Figure 1 visually summarizes the adjusted odds ratios and 95% CIs from the final multivariable LR model. The vertical dashed line indicates an odds ratio of 1.0, representing a null association. This demonstrates that the determinants of care needs vary substantially in magnitude, underscoring the heterogeneous contributions of functional, health-related, and environmental factors within the multivariable model.

### Model Performance

Cross-validated performance (5-fold stratified on the training set) is shown in Table 3. GBDT achieved the highest AUC (mean 0.913, SD 0.007), narrowly ahead of XGBoost (mean 0.909, SD 0.010) and RF (mean 0.907, SD 0.007). All 7 algorithms achieved an AUC of more than 0.85 in model A, indicating that the upstream signal is robust to algorithm choice and not dependent on any single inductive bias.

**Table 3.** Performance of 7 ML algorithms on the held-out test set (N=3024) for model A (primary, no activities of daily living [ADL] or instrumental activities of daily living [IADL]) and model B (benchmark, all features).

Feature set and algorithm	AUC^a^	Sensitivity	Specificity	*F*_1_-score	Brier
Model A					
GBDT^b^ (best, 95% CI)	0.913 (CV)^d^; 0.892 (0.866‐0.918)	0.882	0.768	0.409	0.055
XGBoost^c^	0.909 (CV)^d^	0.864	0.815	—^e^	0.076
Random forest	0.907 (CV)^d^	0.863	0.815	—	0.057
LightGBM^f^	0.903 (CV)^d^	0.832	0.831	—	0.066
Logistic	0.901 (CV)^d^	0.859	0.795	—	0.116
SVM^g^	0.869 (CV)^d^	0.856	0.753	—	0.077
Decision tree	0.855 (CV)^d^	0.856	0.723	—	0.101
Model B					
GBDT (best)	0.978 (CV)^d^	0.996	0.914	0.687	0.036

a AUC: area under the receiver operating characteristic curve.

b GBDT: gradient-boosted decision trees.

c XGBoost: extreme gradient boost.

d CV=mean across 5-fold cross-validation; 95% CI for held-out test set is shown only for the lead (best) algorithm in each feature set. The full table including operating thresholds and calibration is provided in Multimedia Appendix 2.

e Not applicable.

f LightGBM: light gradient boosting machine.

g SVM: support vector machine.

On the held-out test set (n=3024), GBDT (model A) achieved an AUC of 0.892 (95% CI 0.866‐0.918, DeLong method; bootstrap 95% CI 0.870‐0.912), with a PR-AUC of 0.530 (6.10 × the no-skill baseline of 0.087). At the Youden-optimal threshold (0.077), sensitivity was 0.882, specificity was 0.768, *F*_1_-score was 0.41, and MCC was 0.41. Calibration was adequate after isotonic recalibration (Brier score=0.055, calibration slope=0.826, and intercept=−0.295); a slope below 1.0 indicates mild overfitting, with predicted probabilities slightly more extreme than observed risks. The cross-validation-to-test AUC drop of 0.021 confirms reliable cross-validated estimates and minimal generalization error. The train-test AUC gap was 0.071, modestly larger than ideal but small enough to rule out severe overfitting given the consistent CV-test gap. Model A thus met the prespecified criterion of an AUC of 0.80 or more without ADL or IADL.

For comparison, model B achieved an AUC of 0.976 (95% CI 0.962‐0.989) using GBDT on the held-out test set, with a sensitivity of 0.996 at the Youden threshold, a train-test AUC gap of 0.018, and a Brier score of 0.036. The 0.084 AUC gap between models B and A, and the near-perfect sensitivity of model B at the Youden threshold, quantitatively confirm the near-tautological relationship between IADL and the self-reported care-need outcome that motivated the prespecified decision to designate model A as the primary model.

**Figure 2.**
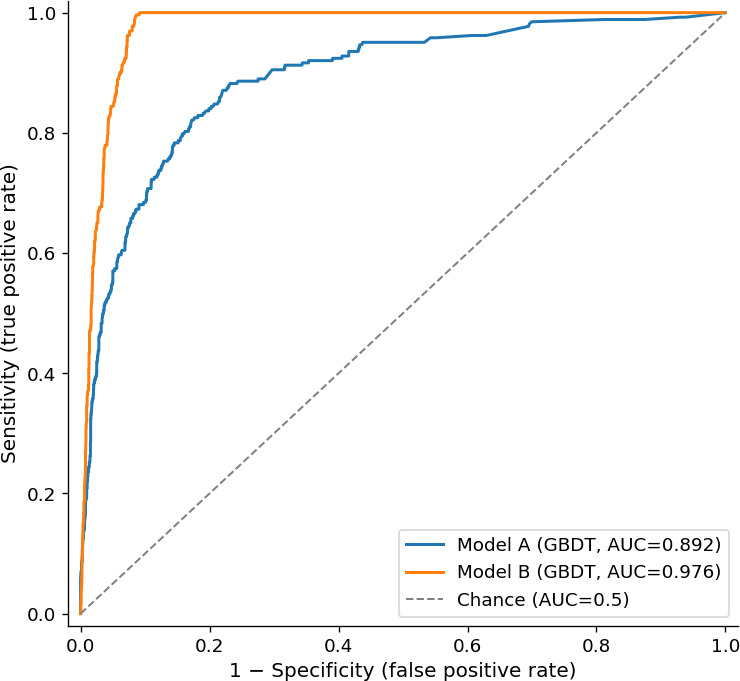
Receiver operating characteristic (ROC) curves for 7 ML models on the held-out test set using model A (primary, no activities of daily living [ADL] or instrumental activities of daily living [IADL]). AUC: area under the receiver operating characteristic curve; SVM: support vector machine; GBDT: gradient-boosted decision trees.

Figure 2 compares the receiver operating characteristic curves of the 7 algorithms on the held-out test set. Boosting-based ensembles (GBDT, XGBoost, and LightGBM) and RF showed similar discrimination, while LR, SVM, and DT achieved slightly lower AUCs.

### Calibration and Clinical Use

**Figure 3.**
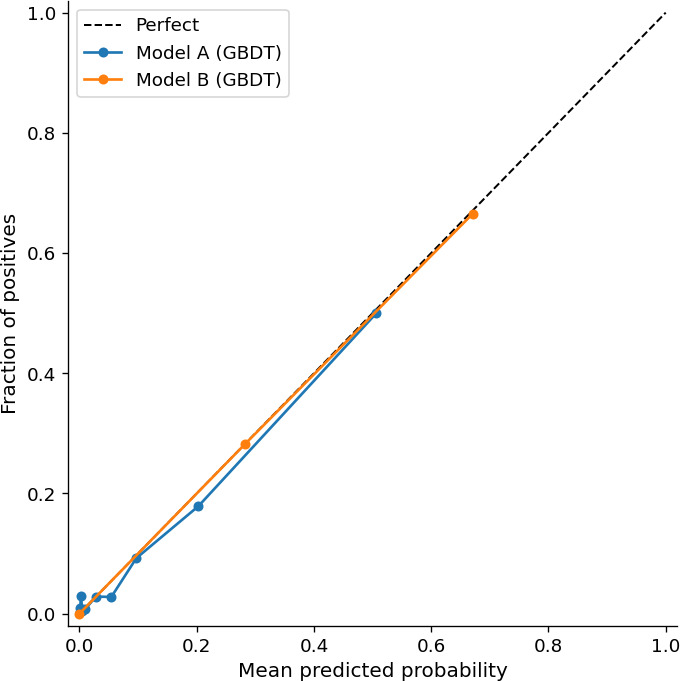
Calibration plot for model A (primary, gradient-boosted decision trees [GBDT]) and model B (benchmark, GBDT) on the held-out test set.

**Figure 4.**
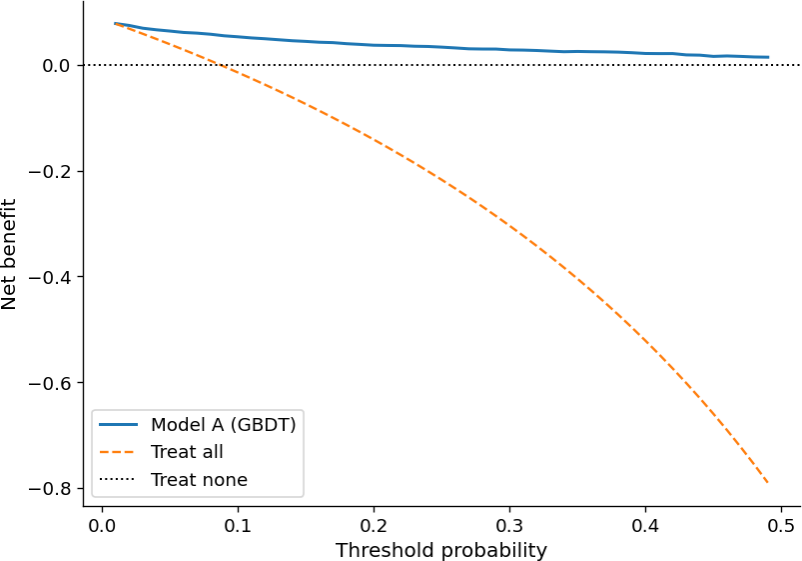
Decision curve analysis for model A on the held-out test set. GBDT: gradient-boosted decision trees.

Calibration plots (Figure 3) show that both model A and model B track the perfect-calibration diagonal closely across the entire range of predicted probabilities observed in the held-out test set (0‐0.7). Decision-curve analysis (Figure 4) demonstrates that model A provides positive net benefit relative to both “treat all” and “treat none” reference strategies across the full clinically plausible range (1%‐50%), suggesting potential use for upstream screening, pending external validation.

### Model Interpretation

**Figure 5.**
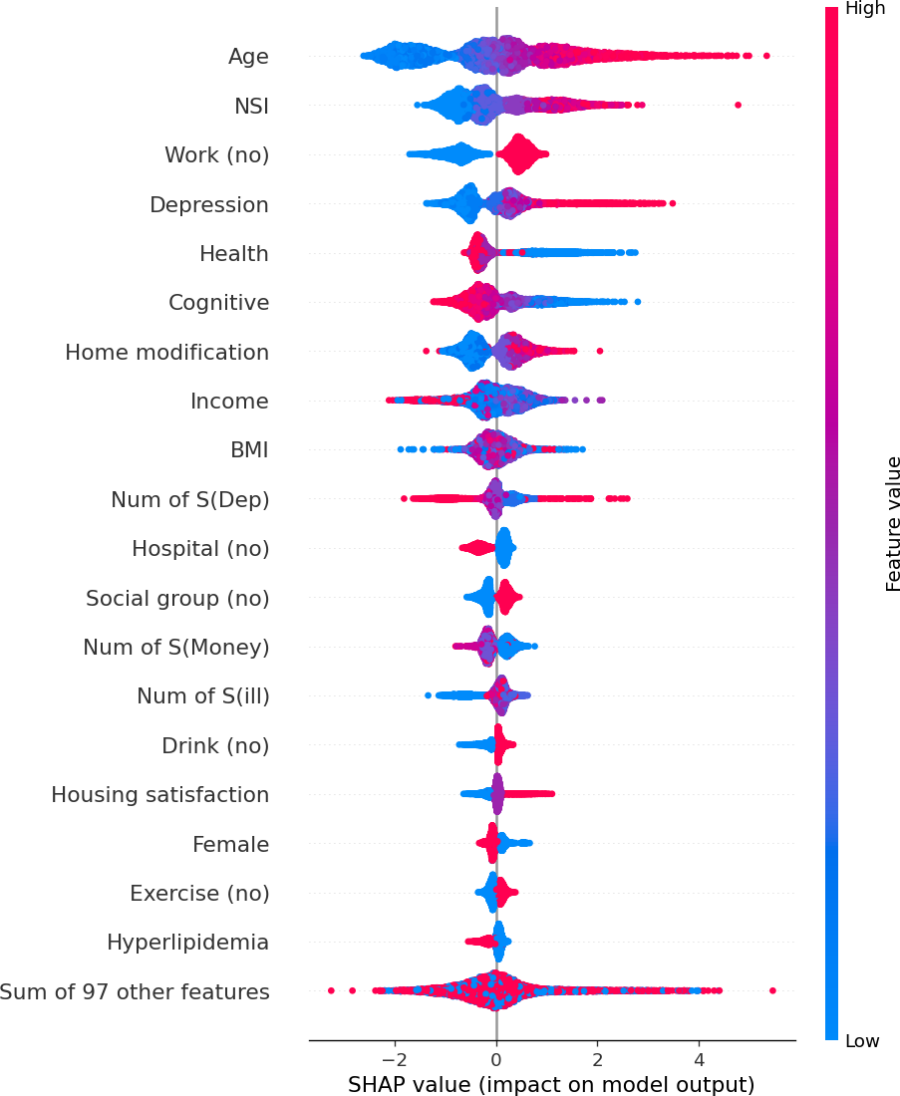
Shapley additive explanations (SHAP) summary plot. All samples and features are displayed, with each row representing a feature and the x-axis representing the SHAP value. Red dots represent higher feature values, while blue and purple dots represent lower feature values. NSI: Nutritional Screening Initiative; Num of S(Dep): number of supporters when feeling depressed; Num of S(ill): number of supporters when ill; Num of S(Money): number of supporters to borrow money from; Income: household income; Health: self-rated health.

**Figure 6.**
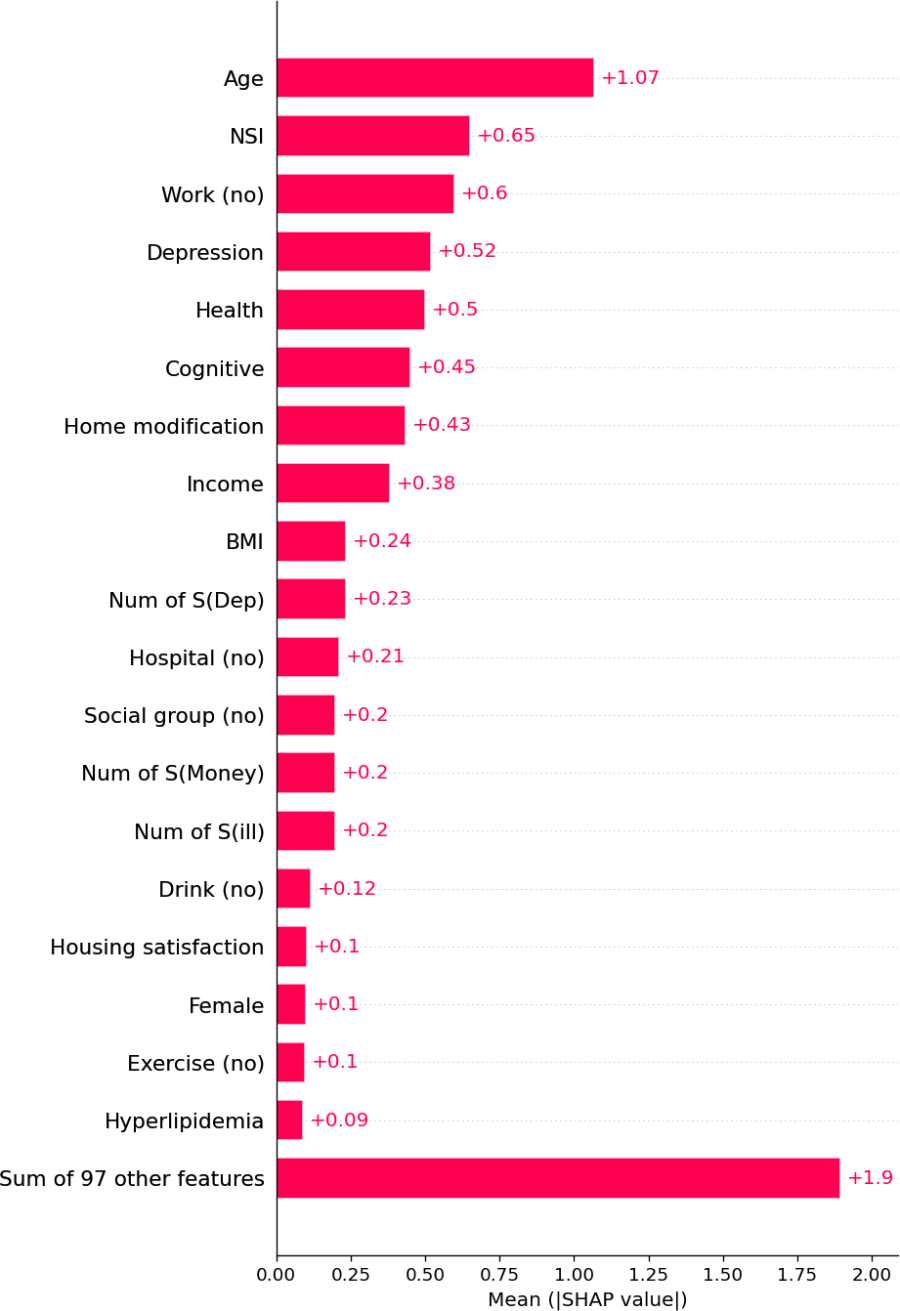
Mean absolute Shapley additive explanations (SHAP) values. Variables are ranked by their mean absolute SHAP values; the y-axis ordering reflects each feature’s global importance to the model. NSI: Nutritional Screening Initiative; Num of S(Dep): number of supporters when feeling depressed; Num of S(ill): number of supporters when ill; Num of S(Money): number of supporters to borrow money from; Income: household income; Health: self-rated health.

Figures 5 and 6 present the results of the SHAP analysis based on the best-performing model (GBDT, model A), with high rank stability across cross-validation folds (Jaccard=0.806 for the top 10), illustrating how individual predictors contribute to model predictions of care needs. Figure 5 shows the distribution of SHAP values for each predictor across individuals, indicating both the direction and magnitude of each variable’s contribution to the predicted probability of care needs. Predictors are ordered according to their overall importance. Figure 6 summarizes the absolute SHAP values, providing a global ranking of feature importance.

SHAP analyses for model A identified age (mean |SHAP| 1.012, SD 0.073; range 0.910‐1.093) as the most influential upstream predictor, followed by nutritional risk (NSI; mean 0.686, SD 0.103; range 0.552‐0.809), employment status (mean 0.628, SD 0.116; range 0.484‐0.769), depressive symptoms (mean 0.530, SD 0.093; range 0.420‐0.606), self-rated health (mean 0.515, SD 0.068; range 0.443‐0.587), cognitive function (mean 0.474, SD 0.069; range 0.398‐0.567), household income (mean 0.432, SD 0.070; range 0.346‐0.524), home modification level (mean 0.364, SD 0.061; range 0.270‐0.407), BMI (mean 0.278, SD 0.032; range 0.244‐0.316), and number of supporters when feeling depressed (mean 0.251, SD 0.056; range 0.176‐0.308). All fold-level intervals excluded zero, supporting the stability of the rankings.

The leading predictors span demographic (age), nutritional (NSI), behavioral (employment), mental (depressive symptoms), subjective-health, cognitive, environmental (home modification), socioeconomic (income), and social-support domains, consistent with the study’s second objective, indicating that care need behaves as a multidimensional construct. SHAP distributions were smoothly graded with no evident clustering, and all directional patterns were clinically interpretable: higher age, NSI, depressive symptoms, and home modification were associated with higher predicted risk; better self-rated health, higher cognitive function, and higher income were associated with lower predicted risk.

### Sensitivity Analyses

For model A, the largest individual ΔAUC drops on removal and retraining were age (−0.005), self-rated health (−0.003), and employment (−0.001). All other top-10 predictors produced |ΔAUC| of 0.002 or less, with 4 (cognitive function, home modification, BMI, and depressive symptoms) showing trivial AUC increases when removed (ΔAUC=0.000‐0.001), reflecting redundancy within the top-10 set rather than weakness of the included variables. No single nonfunctional variable produced a catastrophic drop, indicating a genuinely multidimensional signal (Multimedia Appendix 3).

When the outcome was redefined as 2 or more IADL limitations and/or 1 or more ADL limitations (1411/10,078, 14.0% prevalence, distinct from the primary self-report 875/10,078, 8.7% prevalence), model A achieved an AUC of 0.892 (95% CI 0.871‐0.912), identical to its performance against the original self-reported outcome. This congruence between 2 substantively different outcome operationalizations suggests that model A’s discrimination is not solely an artifact of self-report bias. However, because the composite outcome itself is based on functional impairment, this analysis primarily demonstrates that the upstream predictors are associated with functional limitation and cannot fully exclude measurement bias in the self-reported outcome.

## Discussion

### Principal Findings

This study aimed to identify determinants of care needs among community-dwelling older adults in Korea using a comprehensive set of multidimensional variables and explainable ML techniques. The principal finding is that care needs can be identified with good discrimination (AUC 0.892, 95% CI 0.866‐0.918), adequate calibration (Brier=0.055 and calibration slope=0.826, indicating mild optimism in predicted probabilities), and positive clinical net benefit across the 1% to 50% threshold range using multidimensional nonfunctional features alone (model A). Model A met the prespecified discrimination criterion (AUC≥0.80) without ADL or IADL. Notably, when functional status variables were excluded to identify upstream risk factors, age, nutritional risk, depressive symptoms, self-rated health, employment status, cognitive function, home modifications, and household income emerged as the leading predictors, with high rank stability across folds (Jaccard=0.806). Discrimination is preserved when the outcome is redefined objectively (AUC=0.892), suggesting that the model captures a construct shared between subjective perception and objective functional impairment, though this does not by itself rule out self-report bias in the primary outcome.

The implications of this study are as follows. First, methodologically, the findings demonstrate the strong utility of explainable ML models in gerontological research. By comparing the predictive performance of 7 ML algorithms, the results suggest that ensemble learning methods are highly effective in capturing complex, nonlinear interactions among diverse risk factors inherent in gerontological data. Furthermore, the integration of SHAP values addresses the limitations of ML and provides transparent and interpretable evidence for prioritizing interventions. This study also replicates a recurring lesson in clinical-prediction ML: unusually high AUCs in survey-based studies often reflect outcome-predictor proximity rather than true upstream predictive value. The full-featured model B achieved an AUC of 0.976, but this reflects the near-tautological inclusion of IADL alongside a self-reported care-need outcome [17,18]; reporting model A as the primary result, alongside calibration, decision-curve analysis, and TRIPOD+AI–compliant documentation, provides a more honest characterization of model utility for screening.

Second, this study verified that care needs are multidimensional constructs shaped not only by physical function but also by upstream sociodemographic and environmental determinants across the following domains: demographics; health (eg, functional status, subjective health, nutritional health, mental health, and cognitive health); health behaviors (experience of falls and health-related behaviors); usage (medical use); social domains (participation, support); and the living environment. While previous studies have been limited to medical records or functional status, this study demonstrates that care needs are shaped by a complex interplay of sociodemographic and environmental factors alongside physical function.

### Policy Implications

Based on the study’s findings, several policy and practical implications can be proposed to support successful aging in place among older adults.

First, it is essential to prioritize functional status for immediate service allocation. Consistent with existing long-term care eligibility criteria, functional limitations (IADL and ADL) were identified as the strongest predictors of care needs in model B. This finding validates the current assessment system, which prioritizes physical assistance for individuals with established functional dependence.

Second, targeting hidden-risk groups for preventive interventions is a critical secondary strategy. Model A highlighted the need for a preventive pathway. Even among older adults without severe functional impairments, specific multidimensional factors are associated with elevated care needs. The analysis identified advanced age, nutritional instability, depressive symptoms, cognitive decline, and poor housing conditions as key predictors. Therefore, a secondary pathway could be considered for older adults who are physically independent but exhibit these vulnerabilities. These cross-sectional associations are hypothesis-generating and require longitudinal confirmation before informing preventive deployment.

Third, care services must extend beyond physical support to include integrated lifestyle and environmental management. Specifically, services should encompass nutritional management, psychological counseling for depression, and cognitive training programs. Additionally, the strong predictive power of housing satisfaction and home modification levels underscores the importance of the living environment in aging. Housing interventions, such as barrier-free renovations and environmental adjustments, may be integrated into care strategies; whether such interventions delay the onset of physical dependency requires prospective evaluation [19].

### Limitations

This study had several limitations. First, as a cross-sectional study, it identified associations but could not establish causal relationships between risk factors and the onset of care needs. Longitudinal studies are needed to track these trajectories over time. Specifically, the model identifies older adults whose contemporaneous profile is consistent with current care needs; it has not been validated as a forward-looking incidence-prediction tool. Longitudinal validation using the panel waves of the Korea Senior Survey is a planned next step.

Second, care need was self-reported and was therefore subject to the cultural, psychological, and gendered patterns of need-perception described in the gerontology literature [20]. This study mitigated this concern through (1) sensitivity analysis using an objective composite outcome (results were substantively unchanged: AUC=0.892), and (2) stratification by depressive symptoms (with discrimination preserved across quartiles). Future work should triangulate self-reported need with caregiver-reported and clinician-assessed need.

Third, the model underwent internal validation only (cross-validation and a held-out test split from the same survey); it has not been externally or temporally validated, and the calibration slope of 0.826 indicates that predicted probabilities would likely require recalibration in new settings. Geographic and cross-national validation in independent cohorts (eg, Japanese LTCI claims; the Survey of Health, Ageing and Retirement in Europe; and the China Health and Retirement Longitudinal Study or Chinese Longitudinal Healthy Longevity Survey) are required before clinical or policy deployment. The present model is framed as an evidence-based step toward, not a substitute for, such validation.

Also, the outcome variable, defined as binary care needs, should be expanded in the context of the LTCI system (eg, predicting grades 1‐5 or preventive levels). Future research should apply multiclass classification models to predict specific levels of care needs, thereby enabling more granular and tailored resource allocation.

Finally, hyperparameters were prespecified rather than tuned. This avoids the optimistic bias from data-driven tuning, but the relative performance across algorithms should be interpreted as a comparison under common, nonoptimized settings, rather than as each algorithm’s best achievable performance.

### Conclusions

This study demonstrates that explainable ML models can accurately and transparently predict care needs among older adults using multidimensional survey data. While functional impairment plays a central role, care needs are also shaped by health-related, nutritional, social, and environmental factors. By highlighting the importance of nonfunctional indicators and emphasizing interpretability, this study offers a robust framework for the identification of care needs to inform proactive, community-based aging, and long-term care policies.

## Supplementary material

10.2196/93371 Multimedia Appendix 1 Shapley additive explanations stability analysis with bootstrap 95% CIs.

10.2196/93371 Multimedia Appendix 2 Operating-point performance tables at multiple decision thresholds.

10.2196/93371 Multimedia Appendix 3 Leave-one-feature-out results for the top-10 predictors of model A.

10.2196/93371 Checklist 1 TRIPOD+AI checklist.

## Abbreviations

ADL: activities of daily living
AUC: area under the receiver operating characteristic curve
CES-D-SF: Center for Epidemiologic Studies Depression Scale–Short Form
DT: decision tree
GBDT: gradient-boosted decision trees
IADL: instrumental activities of daily living
LightGBM: light gradient boosting machine
LR: logistic regression
LTCI: Long-Term Care Insurance
MCC: Matthews correlation coefficient
ML: machine learning
NSI: Nutritional Screening Initiative
PR-AUC: precision-recall area under the curve
RF: random forest
SHAP: Shapley additive explanations
SVM: support vector machine
TRIPOD+AI: Transparent Reporting of a Multivariable Prediction Model for Individual Prognosis or Diagnosis+AI
VIF: variance inflation factor
XGBoost: extreme gradient boosting

## References

[1] Jiang M, Li X (2025). Explainable machine learning models predicting the risk of social isolation in older adults: a prospective cohort study. BMC Public Health.

[2] Wang X, Zhang D, Lu L (2025). Development and validation of an explainable machine learning model for predicting the risk of sleep disorders in older adults with multimorbidity: a cross-sectional study. Front Public Health.

[3] Lee SM, Ha HJ (2025). Prediction of depression in the elderly using automated machine learning. J Korea Inf Technol Soc.

[4] Kim JH, Kim HJ, Kim SI, Park BY (2024). Study on creating a depression group discrimination model for the elderly using machine learning and researching factors related to depression. Health Welf.

[5] Suh M, Jung H, Kim J (2024). Development of a fall prediction model for community-dwelling older adults in South Korea using machine learning: a secondary data analysis. J Korean Biol Nurs Sci.

[6] Ren H, Zheng Y, Li C (2025). Using machine learning to predict cognitive decline in older adults from the Chinese Longitudinal Healthy Longevity Survey: model development and validation study. JMIR Aging.

[7] Isaradech N, Sirikul W, Buawangpong N, Siviroj P, Kitro A (2025). Machine learning models for frailty classification of older adults in Northern Thailand: model development and validation study. JMIR Aging.

[8] Fukunishi H, Kobayashi Y (2023). Care-needs level prediction for elderly long-term care using insurance claims data. Inform Med Unlocked.

[9] Song MK, Park Y, Han EJ (2022). Development of prediction model identifying high-risk older persons in need of long-term care. Korean J Appl Stat.

[10] Lu Y, Sato K, Nagai M, Miyatake H, Kondo K, Kondo N (2023). Machine learning–based prediction of functional disability: a cohort study of Japanese older adults in 2013–2019. J Gen Intern Med.

[11] Han SY (2025). A study on multidimensional predictors of frailty in community-dwelling older adults: a decision tree-based approach. Health Soc Welf Rev.

[12] Wiles JL, Leibing A, Guberman N, Reeve J, Allen RES (2012). The meaning of “aging in place” to older people. Gerontologist.

[13] Collins GS, Moons KGM, Dhiman P (2024). TRIPOD+AI statement: updated guidance for reporting clinical prediction models that use regression or machine learning methods. BMJ.

[14] Chawla NV, Bowyer KW, Hall LO, Kegelmeyer WP (2002). SMOTE: synthetic minority over-sampling technique. J Artif Intell Res.

[15] Vickers AJ, Elkin EB (2006). Decision curve analysis: a novel method for evaluating prediction models. Med Decis Making.

[16] Lundberg SM, Lee SI A unified approach to interpreting model predictions.

[17] Rudin C (2019). Stop explaining black box machine learning models for high stakes decisions and use interpretable models instead. Nat Mach Intell.

[18] Ramadan B, Liu MC, Burkhart MC, Parker WF, Beaulieu-Jones BK (2025). Diagnostic codes in AI prediction models and label leakage of same-admission clinical outcomes. JAMA Netw Open.

[19] Cha SM (2025). A systematic review of home modifications for aging in place in older adults. Healthcare (Basel).

[20] Zhang J, Zhang Y, Bennett MR (2025). Spousal characteristics and unmet care needs: a longitudinal national study of adults aged 50 and over in England. Soc Sci Med.

[21] Korea Institute for Health and Social Affairs (KIHASA).

